# MosquitoSong+: A noise-robust deep learning model for mosquito classification from wingbeat sounds

**DOI:** 10.1371/journal.pone.0310121

**Published:** 2024-10-30

**Authors:** Akara Supratak, Peter Haddawy, Myat Su Yin, Tim Ziemer, Worameth Siritanakorn, Kanpitcha Assawavinijkulchai, Kanrawee Chiamsakul, Tharit Chantanalertvilai, Wish Suchalermkul, Chaitawat Sa-ngamuang, Patchara Sriwichai

**Affiliations:** 1 Faculty of ICT, Mahidol University, Nakhon Pathom, Thailand; 2 Bremen Spatial Cognition Center, University of Bremen, Bremen, Germany; 3 Institute of Systematic Musicology, University of Hamburg, Hamburg, Germany; 4 Faculty of Tropical Medicine, Mahidol University, Bangkok, Thailand; University of Saskatchewan College of Agriculture and Bioresources, CANADA

## Abstract

In order to assess risk of mosquito-vector borne disease and to effectively target and monitor vector control efforts, accurate information about mosquito vector population densities is needed. The traditional and still most common approach to this involves the use of traps along with manual counting and classification of mosquito species, but the costly and labor-intensive nature of this approach limits its widespread use. Numerous previous studies have sought to address this problem by developing machine learning models to automatically identify species and sex of mosquitoes based on their wingbeat sounds. Yet little work has addressed the issue of robust classification in the presence of environmental background noise, which is essential to making the approach practical. In this paper, we propose a new deep learning model, MosquitoSong+, to identify the species and sex of mosquitoes from raw wingbeat sounds so that it is robust to the environmental noise and the relative volume of the mosquito’s flight tone. The proposed model extends the existing 1D-CNN model by adjusting its architecture and introducing two data augmentation techniques during model training: noise augmentation and wingbeat volume variation. Experiments show that the new model has very good generalizability, with species classification accuracy above 80% on several wingbeat datasets with various background noise. It also has an accuracy of 93.3% for species and sex classification on wingbeat sounds overlaid with various background noises. These results suggest that the proposed approach may be a practical means to develop classification models that can perform well in the field.

## Introduction

Mosquito vector-borne diseases such as malaria, dengue, and Zika pose some of the most serious public health burdens in tropical and sub-tropical countries [1]. Due to the ongoing climate change, urbanization, and other global changes, the geographical range of vector-borne diseases is expected to further expand [2, 3]. In order to assess risk, effectively target public health interventions, and monitor the effectiveness of vector control efforts, accurate information about mosquito vector population densities is needed. Since different species of mosquitoes transmit different diseases, we require not only overall mosquito population density estimates, but also estimates by species.

The traditional and most common approach for adult mosquito vector monitoring is to deploy collection methods and then manually count and identify the species of the mosquitoes caught. Commonly used approaches include traps that contain one or more attractants such as light, CO_2_, heat, or odor [4, 5], cow bait tents [6], and human landing catch [7]. These approaches are highly labor-intensive in terms of deployment, as well as classification of the mosquitoes caught. Because of the labor-intensive nature, they are typically used only for occasional surveys. In addition, because of the effort required to deploy the collection methods, they are primarily used with limited coverage and thus only to estimate the relative populations of different species, rather than population densities, which would be highly valuable information to have. Thus, there is need for an alternative approach that could enable accurate estimation of population densities of the variety of mosquito species present on a continuing basis and at low cost.

It has been long shown that the different mosquito species have different wingbeat audio signatures [8], enabling them to recognize each others’ species and sex [9, 10]. Many studies have developed machine learning models to automatically identify species and sex of mosquitoes based on their wingbeat sounds. Several works have proposed to extract features from the wingbeat sounds, such as fundamental frequency [11] and MFCC [12], for the classification. Other researchers have suggested to extract features after processing the mosquito sound in a way that mimics the sound processing in the mosquito antenna [13]. Some researchers have found that the fundamental frequency of wingbeat seems to be insufficient to differentiate between mosquito species [14]. Recently, researchers have turned to employ the popular deep learning models for image classification [15–17] to extract features from the spectrogram representations of the wingbeat recordings for species and sex identification [18–22]. The utilization of spectrograms with deep learning models is similar to the approaches to other bioacoustic classification problems such as classification of birds [23–25], bats [26], elephants [27], fish [28] and insects [29]. However, recent work has suggested that use of spectrograms may overlook some details that are important for fine high-resolution discriminations [30, 31]. Training a model to discover and extract features from raw audio signals may overcome these issues. For example, Varma et al. [32] showed that a SincNet model [33] operating on raw audio outperformed a CNN operating on a Mel-spectrogram representation of the same data for the task of distinguishing between crickets and katydids. Another recent study has demonstrated the potential of a 1D-CNN model and a 1D-CNN with LSTM model for mosquito classification based on raw audio waveforms [34].

Since the acoustic methods are sensitive to the quality and type of the microphones, as well as background noise present under field conditions, several approaches have been proposed to address these issues. Wavelet transforms have been applied to transform from audio waveforms into spectrograms before training a CNN model for mosquito detection [21]. Later, this work was evaluated with the existing Humbug Zooniverse [35] dataset, which contains wingbeat recordings captured using smartphones in noisy field environments. Results showed promising performance with true positive and true negative rates of 89% and 97%, respectively, for the task of distinguishing between wingbeat sounds and noise [19]. Another study also proposed to use Mel-frequency spectrograms with a CNN model to identify mosquito species and evaluated the model using the recordings from the field-captured mosquitoes in cups with background noise [18]. The results showed an average classification accuracy of only 60%, which could be due to the class imbalance and the limited number of labeled examples. Apart from utilizing spectrograms, a recent study has developed a low-cost acoustic sensor to monitor and classify mosquitoes in a field environment [11]. The fundamental frequencies extracted using Fast Fourier Transform (FFT) and a simple rule-based model were used for counting mosquitoes. Band-pass filtering and smoothing functions in a scrolling window were used to alleviate the impact of background noises. Later, the same approach was evaluated in a different area with help from specialists in labeling the species and sex of mosquitoes in the traps [36]. They found that the fundamental frequency alone could not distinguish different species due to the overlapping frequencies and the effect of environmental noise.

Another research direction is to use an optical sensor to capture light fluctuations when a mosquito flies across the sensor and then synthesize pseudo wingbeat sounds. Such synthesized wingbeat sounds are unaffected by wind noise and ambient sounds, and their potential has been demonstrated for mosquito species and sex identification [14]. A recent study has demonstrated in a controlled environment that such optical sensors can achieve high accuracy in sex classification, but not in species and sex classification [37]. Another study has proposed to convert the synthesized wingbeats to spectrograms and use CNN models to identify mosquito species [38]. This optical sensing technology should be considered as complementary to acoustic sensors. While the optical sensors are unaffected by audio background noise, they are sensitive to optical background noise such as ambient light and airborne dust. The same techniques for classification using acoustic data can also be applied to the wingbeat sounds synthesized from optical sensors.

In order to train a model to be robust to environmental noise, a potential solution is to include the noise during the model training, which has shown promising results in bird classification [24]. However, the process of gathering mosquito wingbeat sounds with noises from the real-field environment is challenging. A more practical alternative is to collect the noises from the field and overlay them with wingbeat sounds collected in controlled environments. This approach also enables us to create variations of the background noises in the wingbeat sounds that can be used to train the classification model to be more robust to such noises (i.e., data augmentation) and that can be used to evaluate the models under varying noise conditions. In the fields of computer vision and audio classification, data augmentation has been shown to help reduce the overfitting problem when the amount of data is limited [39, 40].

In this paper, we propose a new deep learning model to identify the species and sex of mosquitoes from raw wingbeat sounds in such a way that it is robust to the environmental noise and the relative volume of the mosquito’s flight tone. The proposed method that we call MosquitoSong+ extends the existing MosquitoSong 1D-CNN model [34], by making adjustments to its architecture and introducing two data augmentation techniques during model training: noise augmentation and wingbeat volume variation. To evaluate our approach in a variety of settings, we carry out evaluation experiments using several datasets. Two datasets contain pure wingbeat sounds: the HumBugDB dataset [35] and indoor recordings collected in our previous work [34]. For the current study, we collected a third dataset in an outdoor urban setting so that it contains wingbeat sounds with background noise. We also use two noise datasets: noise recordings from HumBugDB and noise that we recorded in an outdoor urban environment. The noise datasets are used to simulate a noisy environment by overlaying them on the two pure wingbeat datasets for the purposes of augmentation and testing. Our main contributions are as follows:

- We show that the MosquitoSong+ model outperforms the previous MosquitoSong model for species and sex classification on wingbeats with synthesized noise.
- We demonstrate model generalizability by showing that MosquitoSong+ has very good performance (accuracy above 0.8) for species classification across a variety of datasets: indoor wingbeats with noise overlay, HumBugDB wingbeats with noise overlay, and outdoor recordings.
- We show that MosquitoSong+ has excellent performance for for the harder problem of both species and sex classification on the indoor recordings (average accuracy 0.933) under a wide variety of simulated background noise, but that due to data limitations performance on outdoor recordings is not a good.
- In addition, all experiments show robustness to the volume of the wingbeat sounds relative to the background noise.

## Materials and methods

### Datasets

#### Indoor wingbeat recordings (W-INDOOR)

The mosquito wingbeat sounds were previously collected from the laboratory of the Medical Entomology Department of the Faculty of Tropical Medicine at Mahidol University [34]. The research has been approved by the Institutional Review Board of the Faculty of Tropical Medicine, Mahidol University (FTM-ACUC 030/2020). The dataset consists of recordings of laboratory strains of four mosquito species: *Aedes aegypti*, *Aedes albopictus*, *Anopheles dirus*, and *Culex quinquefasciatus* from both males (M) and females (F). Each mosquito was individually put into a small cylindrical net cage (8 cm width and 12 cm height). A condenser (Studio Behringer ECM8000 measurement) and a low-cost (Primo EM172) microphone were used to record the wingbeat sounds at 24-bit depth and 96 kHz sampling rate. The raw recordings were processed by extracting only the periods containing wingbeat sounds. These wingbeat sounds were then split into the 300-ms epochs with 150-ms overlap for training and evaluating the classification model. Table 1 summarizes the total duration (in seconds) of the wingbeat recordings and the number of 300-ms epochs of the wingbeat sounds from each species and sex after the split.

**Table 1:** The total duration (in seconds) of the wingbeat recordings and the number of 300-ms epochs from each species and sex for the three datasets. For the HumBug dataset, mosquitoes of some sexes are not available and the sexes of some species are not indicated.

Species	Sex	Indoor		Outdoor		HumBugDB	
		Duration	Epochs	Duration	Epochs	Duration	Epochs
*Ae. aegypti*	F	197	1, 599	86	405	84	500
	M	198	1, 673	10	37	0	0
	Unk	0	0	0	0	0	0
*Ae. albopictus*	F	233	1, 808	67	275	11	41
	M	256	2, 020	31	147	5	14
	Unk	0	0	0	0	19	70
*An. dirus*	F	152	1, 257	54	316	129	500
	M	87	907	81	424	0	0
	Unk	0	0	0	0	0	0
*Cx. quin*	F	305	2, 203	0	0	0	0
	M	456	3, 023	83	420	0	0
	Unk	0	0	0	0	100	500
**Total**		1,884	14,490	412	2, 024	346	1, 625

Unk refers to unknown sex, and *Cx. quin* refers to *Cx. quinquefasciatus* species.

#### Outdoor wingbeat recordings (W-OUTDOOR)

In addition to the indoor recordings, we collected wingbeat sounds from an urban environment. This involved placing live adult mosquitoes inside a small netted cylinder (measuring 6 cm × 8 cm) equipped with a low-cost Primo EM172 microphone. The cylinder was positioned approximately 5 cm above a Biogents BG-counter 2 trap designed for outdoor mosquito monitoring. Our objective was to record the wingbeat sounds of mosquitoes in their natural surroundings while considering outdoor noise and the trap’s fan noise. We maintained the same audio configuration in collecting data for the four mosquito species listed above. To accommodate the mosquitoes’ diurnal and nocturnal activity patterns, we recorded them during specific time periods. For day-active *Aedes* mosquitoes, recordings were made during the daytime. In contrast, recordings were conducted from dusk to the following morning for the *Anopheles* and *Culex* species, which are active during the evening and night. All recordings were captured at a sampling rate of 96 kHz and a depth of 24 bits. Table 1 summarizes the data.

#### Environmental noise recordings (N-OUTDOOR)

In the same urban environment as the outdoor setting, we also recorded environmental noises, comprising vehicles, animals (cats and dogs), and human activity (watering with a garden hose, sweeping, and cutting grass) using a condenser microphone. Since we are interested in the possibility of using our classifiers in conjunction with mosquito traps, the classifiers need to be robust to the noise of the fan that is used in such traps. Thus, we also collected the fan noises from a CDC light trap model 512 (John W. Hock, Gainesville, FL) and a miniature light trap Model 2836BQ (BioQuip, Rancho Dominguez, CA).

#### HumBugDB dataset (W-HUMBUG)

To further evaluate the generalizability of the proposed model, we utilized the existing mosquito wingbeat and noise recordings from the HumBugDB public dataset [35]. As our indoor and outdoor recordings contain wingbeat recordings from four species, we only used the subset of the recordings from these species to facilitate the performance comparison across different datasets.

- ***Aedes aegypti*** The recordings were gathered from wild *Aedes aegypti* mosquitoes sampled in Tanzania. These mosquitoes were collected and recorded in sample cups using the Telinga EM23 field microphone at a sampling rate of 44.1kHz and a 24-bit depth. According to the given metadata, only female *Aedes aegypti* appear in the total of 1322.4 seconds of recordings.
- ***Aedes albopictus*** Laboratory cultures raised at the US Center for Diseases Control and Prevention were recorded using smartphones with 8 kHz sampling rate and 24-bit depth. We selected those recordings consisting of only a single mosquito, resulting in 33.2 seconds of wingbeat sounds. The sex labels of more than half of the audio signals were not available.
- ***Anopheles dirus*** Wild mosquitoes sampled at a mosquito monitoring site in Thailand were brought to a laboratory and recorded using a setup similar to the one used to record the *Aedes aegypti* sounds. A total of 909.8 seconds of recordings are provided.
- ***Culex quinquefasciatus*** Laboratory cultures at the University of Oxford, UK, were recorded using the same setup as for *Aedes aegypti*. Since there are no labels indicating the number of mosquitoes in a cup, we manually selected recordings with a single mosquito. In addition, the sex of the mosquitoes was not indicated.

Since sex annotations were not available for some species, this dataset can only be used to evaluate the species classification. The total duration (in seconds) and the total number of epochs after splitting are shown in Table 1.

*Environmental noises (N-HUMBUG).* In the recordings of mosquito wingbeats, there are segments labeled as background noise that do not overlap with the wingbeat sounds. These noises come from human activities, including human speech and tapping sounds caused by handling containers. We used these noise segments from two different recording setups—one conducted in Thailand using a Telinga EM23 microphone, and the other in the UK using a Telinga EM23 microphone and a phone. The total duration of the noise recordings used is summarized in Table 2.

**Table 2:** The total duration of the N-HUMBUG and N-OUTDOOR datasets from each category and recording device.

**N-OUTDOOR**	
**Environmental Noise (by category)**	**Duration (s)**
Animals	14.59
Fan	6.00
Human activities	124.38
Vehicles	19.07
Total	164.04
**N-HUMBUG**	
**Environmental Noise (by device)**	**Duration (s)**
Telinga EM23 (Thailand)	188.07
Telinga EM23 (UK)	20.99
Phone (UK)	56.70
Total	265.76

Each audio file has a unique ID and date when it was recorded. Some of these files were parts of the same longer recording, meaning that they probably captured the wingbeat sound of the exact same individual mosquito. Therefore, we randomly selected only one segment from those longer recordings. This is to ensure we obtained a variety of 300-ms segments from various mosquitoes.

#### Data preprocessing

Since the proposed approach is intended to be used with low-cost IoT devices with relatively low computing power, the whole dataset of both mosquito wingbeats and noise was downsampled before being used in the model training and evaluation. A recent study demonstrated that a deep learning model can achieve a classification performance on wingbeat recordings with a sampling rate of 8 kHz and 16-bit depth similar to that achieved on the same recordings with a sampling rate of 96 kHz with a 24-bit depth [41]. We, therefore, downsampled wingbeat sounds from all datasets as well as noise recordings to have a sampling rate of 8 kHz with 16-bit depth. So that we could use the entire bit depth without any clipping, the recordings were normalized using the maximum absolute value of the amplitude from all wingbeat recordings. The recording of each species from the HumBugDB dataset was divided by the maximum absolute amplitude of the species due to a variety of collection methods and sources. On the other hand, since the same recording setting was used for each of the entire indoor clean wingbeat and outdoor datasets, the normalization was done using the maximum absolute value from the entire dataset.

### Noise overlay simulation

We simulated epochs of noisy wingbeat sounds, **x**_nw_, with the following equation:

$$\begin{array}{c} x_{\text{nw}}=G\cdot x_{\text{w}}+x_{\text{n}} \end{array}$$ (1)

where **x**_w_ and **x**_n_ are randomly selected epochs of the original wingbeat and the noise recordings, respectively. Both **x**_w_ and **x**_n_ are of the same size: $R^{s\times f_{s}}$. The value of *s* represents the duration of each epoch in seconds, and *f*_*s*_ denotes the sampling rate in Hz, which depends on the model architecture that we will discuss later. The gain factor *G* is a parameter that is used to adjust the amplitude of mosquito sounds relative to the background noises. Essentially, this factor can be interpreted as varying the distance between the mosquitoes and the recording microphone. When *G* = 1, the original wingbeat recordings were used, without any adjustments. In case *G* is between 1 and 2, a fairly audible mosquito sound can be heard even in the presence of continuous background noise. However, the same mosquito sound becomes inaudible in the presence of obtrusive sounds, such as bird calls and car brakes.

**Fig 1:**
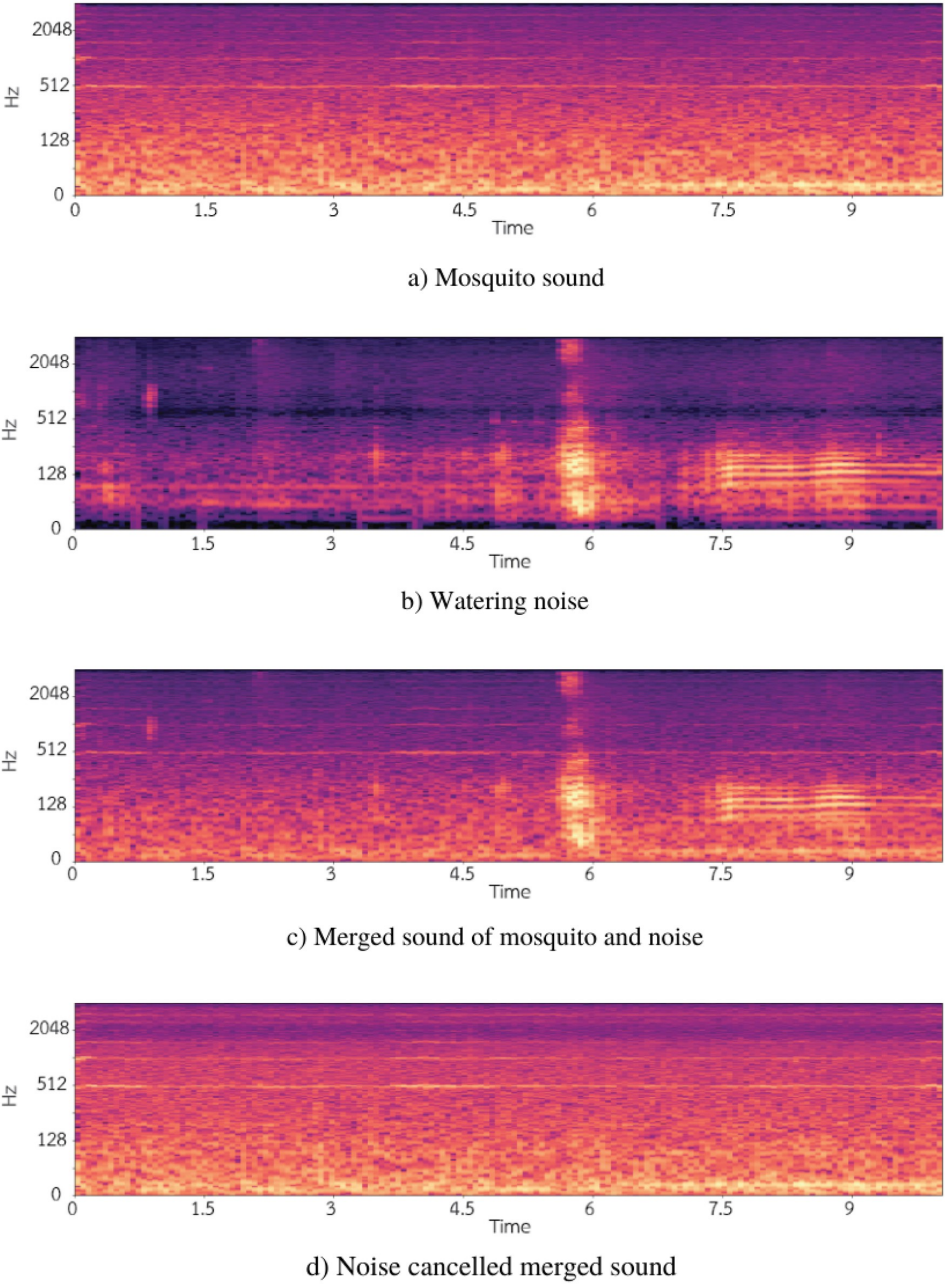
Example spectrograms of the noise-overlaid wingbeat sounds. The spectrograms of a 10-s recordings of (a) a female *Aedes aegypti* wingbeat, (b) a watering noise, and (c) a wingbeat merged (or overlaid) with watering noise. The frequency components of the wingbeat sounds (e.g., around 512 Hz) are still present in the spectrogram of the noise-overlaid sound.

Fig 1 shows an example of the spectrogram of the noise-overlaid wingbeat sound. The mosquito produces a relatively steady, harmonic sound (parallel lines in Fig 1a). The recorded watering noise (Fig 1b) is partly impulsive (vertical line at 6 seconds) and partly harmonic (parallel lines from 7.5 to 10 seconds). When merging the two (Fig 1c), both the mosquito and the watering can be seen in the spectrograms and heard in the audio.

### MosquitoSong+ model

Our proposed model, named *MosquitoSong+*, is an extension of the previously proposed *MosquitoSong* [34] deep learning model for mosquito species and sex classification from low-sample-rate raw audio signal without noise. The MosquitoSong+ model uses a modified architecture, as well as two data augmentation techniques to add variations of background noise and gain factors to the wingbeat sounds during the model training.

#### Model architecture

**Fig 2:**
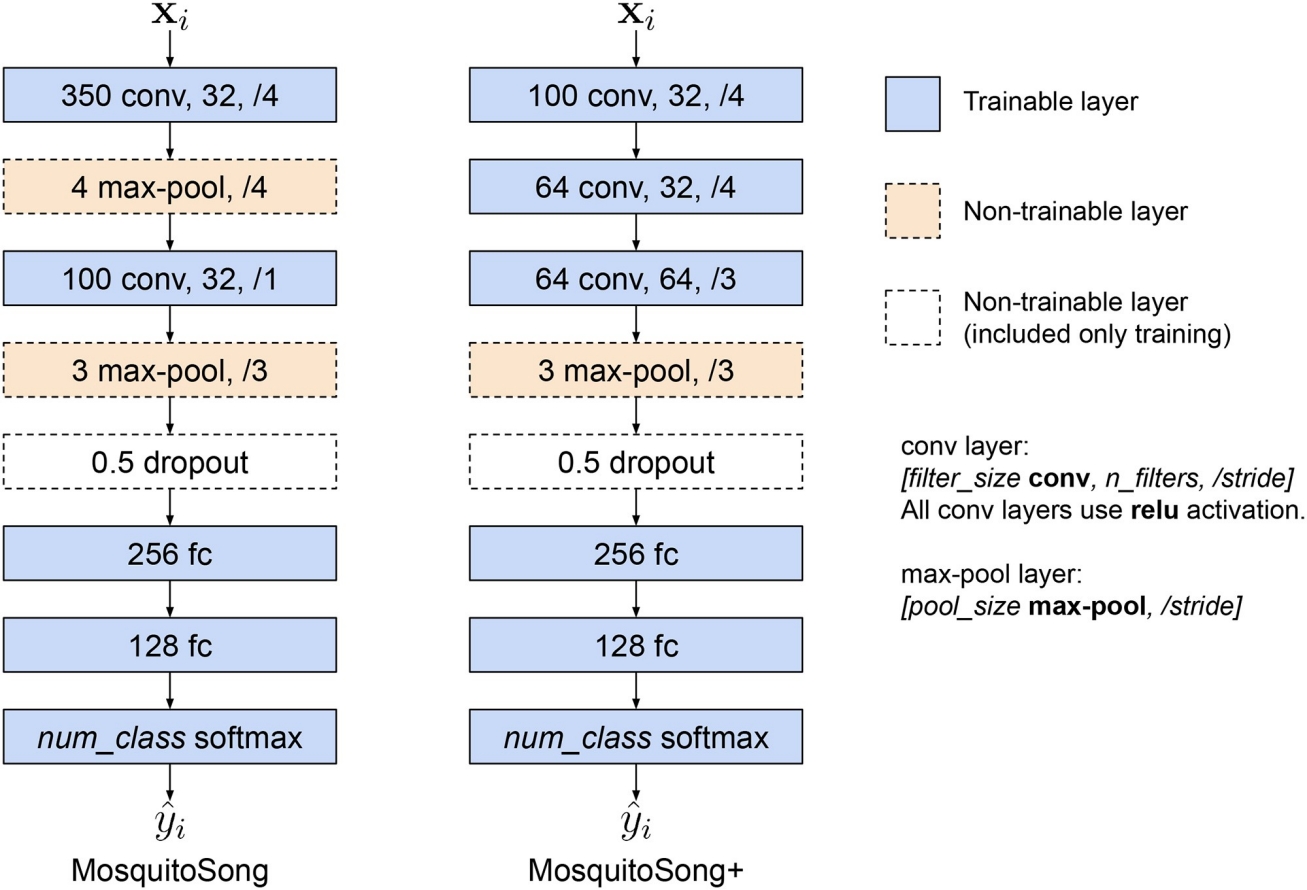
Comparison between the model architectures of the MosquitoSong (left) and MosquitoSong+ (right). The value of *num*_*class* in the final layer of both models varies depending on the number of species and/or sex intended for training.

In the MosquitoSong+ model, we replaced the initial three layers of the MosquitoSong model with three new 1-D convolutional layers, as illustrated in Fig 2. Our findings indicate that this stack of convolutional layers exhibits improved generalizability, primarily attributable to its learnable weights for downsampling, in contrast to the previous approach that relied on simple statistical summarization with the maximum value.

The model receives an epoch of input sounds and determines the species and sex of a mosquito. There are eight classes in this study, corresponding to four mosquito species: *Aedes aegypti*, *Aedes albopictus*, *Anopheles dirus*, and *Culex quinquefasciatus* for both males and females. Each input passes through two blocks of 1-D convolution and a 1-D max-pooling layers, two fully-connected layers, and a final softmax layer to predict a probability value between 0 and 1 for each output class.

Formally, suppose there are *N* epochs of input sounds: {**x**_1_, …, **x**_*n*_}, where $x_{i}\in R^{s\times f_{s}}$, *s* is the duration in seconds of each epoch, and *f*_*s*_ is the sampling rate in Hz of the input sound. The model determines the species and sex for all epochs, resulting in *N* predicted classes $\left\{{\hat{y}}_{1},\ldots ,{\hat{y}}_{N}\right\}$, where ${\hat{y}}_{i}$ is the predicted class of **x**_*i*_, and ${\hat{y}}_{i}\in \left\{0,1,2,\ldots ,7\right\}$ corresponding to each species and sex mentioned earlier. In this study, *s* and *f*_*s*_ are 0.3 seconds (or 300 milliseconds) and 8000 Hz, resulting in an input size of 2400 values.

#### Model training with data augmentation

Our technique trains the model end-to-end via minibatch gradient descent, equipped with data augmentation. Such data augmentation helps to produce new training examples from the original ones for *every* training epoch. The weighted cross-entropy loss is used to minimize the class imbalance problem. The model is trained for 1000 epochs with the Adam optimizer [42] using a learning rate of 0.0001. The best performing model based on the validation set is used.

The two data augmentation techniques used are noise augmentation and wingbeat volume variation:

- **Noise augmentation.** An epoch of the environmental noise is randomly sampled from the pool of different types of noises, which is then added to the wingbeat sounds. This technique helps introduce new wingbeat sounds with different background noises, such that the model is better at learning the patterns of the wingbeat sounds. To prevent the model from overfitting to the environmental noises, the original pure wingbeat sounds are also included during the training with a probability of 10%. As a result, 90% of the training set in each training epoch will have background noise and the other 10% will not.
- **Wingbeat volume variation.** A gain factor (i.e., *G* in Eq 1) randomly selected from the range of 1 to 2 is used to multiply the wingbeat sounds before the environmental noise addition. As the mosquitoes are not flying at the same distance from the microphones all the time, it is more realistic to vary the amplitude of the wingbeat sounds relative to the background noise. This technique helps generate realistic dynamics of the mosquitoes flying past the microphones.

These techniques are also used in conjunction with the data augmentation techniques used in the previous model [34], which are Gaussian noise addition, time shifting, and amplitude variation:

- **Gaussian noise addition.** An epoch of Gaussian noise multiplied with a factor randomly sampled from a range of 0.001 to 0.01 is added to an input sound.
- **Time shifting.** An input sound is randomly shifted along the time axis. The shifting amount is uniformly sampled from a range of ±10% of the 300-ms epoch.
- **Amplitude variation.** An input sound is multiplied with a random amplitude factor of 1/4 to 4 to reduce or increase the volume with a range of −12 to 12 dB.

With these data augmentation techniques, we can train the model to be robust to the variation of background noise and the dynamics of the wingbeat sounds from the flying mosquitoes. Our code is publicly available at https://github.com/akaraspt/mosquitosongp.

## Results

### Performance metrics

Per-class precision (PR), per-class recall (RE), per-class F1-score (F1), macro-averaged F1-score (MF1), and overall accuracy (ACC) were used to evaluate the proposed approach. The per-class metrics were computed by considering one species and sex as a positive class, and all others combined as a negative class. The ACC and MF1 were computed as follows:

$$\begin{array}{c} \text{ACC}=\frac{\sum_{c=1}^{C}{\text{TP}}_{c}}{N} \end{array}$$ (2)

$$\begin{array}{c} \text{MF1}=\frac{\sum_{c=1}^{C}{\text{F1}}_{c}}{C} \end{array}$$ (3)

where TP_*c*_ is the true positives of class *c*, *F*1_*c*_ is per-class F1-score of class *c*, *C* is the number of classes (i.e., the number of mosquito species and sex), and *N* is the total number of test examples. The non-parametric Mann-Whitney U Test (alpha value = 0.05, one-sided test) was also used to examine the statistical significance when we compared the performance metrics between two settings.

### Experiment 1: Model improvement—Classification of species and sex under noise overlay simulation

#### Experimental setup

The goal of this experiment is to evaluate the effectiveness of our extension to the previous MosquitoSong model [34]. The experimental setup is similar to the earlier work. Our proposed approach was evaluated using stratified 10-fold cross-validation under noise overlay simulation, utilizing the W-INDOOR and N-OUTDOOR datasets. In particular, the wingbeat sounds from each species and sex were split chronologically into 10 folds. For each fold, one part was used as the test set, and the remaining parts were used for the training and the validation sets. This process was repeated 10 times, yielding a total of 10 models that were trained and evaluated to get the predictions from all folds. As the proposed model would eventually be deployed with a low-cost microphone, only the wingbeat recordings from the low-cost microphone were used in the test set (i.e., no wingbeat recordings from the condenser microphone).

We made sure that there was no overlap between the training, validation, and test sets in each fold for both the wingbeat and environmental noises. Additionally, we maintained the original wingbeat sounds in the test set, such that there were wingbeats both with and without background noise. This allowed for testing whether the model was overfitted to the environmental noise. It is also realistic to assume that in the real environment, there would be periods of wingbeat without background noise.

To simulate situations in which mosquitoes fly close to or far away from the microphones, we also applied different gain factors (*G*): 1, 1.5 and 2 to the wingbeat sounds in the test set for both simulations. By applying three different *G* values to the test sets, the stratified 10-fold cross-validation was repeated three times. The predictions from all folds and the three *G* values (30 in all) were combined and used to compute the performance metrics, which are discussed in the next section.

#### Impact of environmental noise

The previous MosquitoSong model [34] was tested with and without the presence of environmental noise (see Table 3). We observed a significant drop in all performance metrics in the presence of environmental noise. The overall ACC/MF1 reduced from 0.908/0.906 to 0.862/0.856 (*p* = 0.0003). This shows that environmental noise has a significant impact on the model performance, and should always be considered in the model evaluation.

**Table 3:** Comparison between MosquitoSong+ (proposed model) and MosquitoSong (previous model) in terms of overall accuracy (ACC), macro-averaging F1-score (MF1), and per-class F1-score. These performance metrics were computed by combining the test sets from three stratified 10-fold cross-validations, each corresponding to the application of one of the three gain factors (*G*): 1, 1.5, and 2. The numbers in bold indicate the highest performance metrics of all methods (excluding the first row that was evaluated with the original recorded wingbeat sounds without noise).

Model	Noise Simulation	ACC	MF1	Per-class F1-score								
				*Ae. aegypti*		*Ae. albopictus*		*An. dirus*		*Cx. quin*		Test Examples
				F	M	F	M	F	M	F	M	
MosquitoSong [34]	No	0.908	0.906	0.929	0.841	0.922	0.912	0.889	0.914	0.960	0.885	6, 000
MosquitoSong [34]	Yes	0.862	0.856	0.884	0.798	0.874	0.852	0.814	0.840	0.929	0.853	36, 000
MosquitoSong+	Yes	**0.918**	**0.911**	**0.924**	**0.862**	**0.908**	**0.949**	**0.881**	**0.886**	**0.981**	**0.896**	36, 000

*Cx. quin* refers to *Cx. quinquefasciatus* species.

#### Performance gain from the proposed model

As shown in Table 3, our MosquitoSong+ model achieved significantly better classification performance (ACC/MF1 = 0.918/0.911) than the MosquitoSong model (ACC/MF1 = 0.862/0.859), (*p* = 9*e* − 4). Additionally, per-class F1-scores indicate that the MosquitoSong+ model demonstrated better performance across all species and genders as compared to the MosquitoSong model. This strongly suggests that the proposed model architecture and data augmentation techniques are effective in reducing the impact of environmental noise on classification performance.

**Fig 3:**
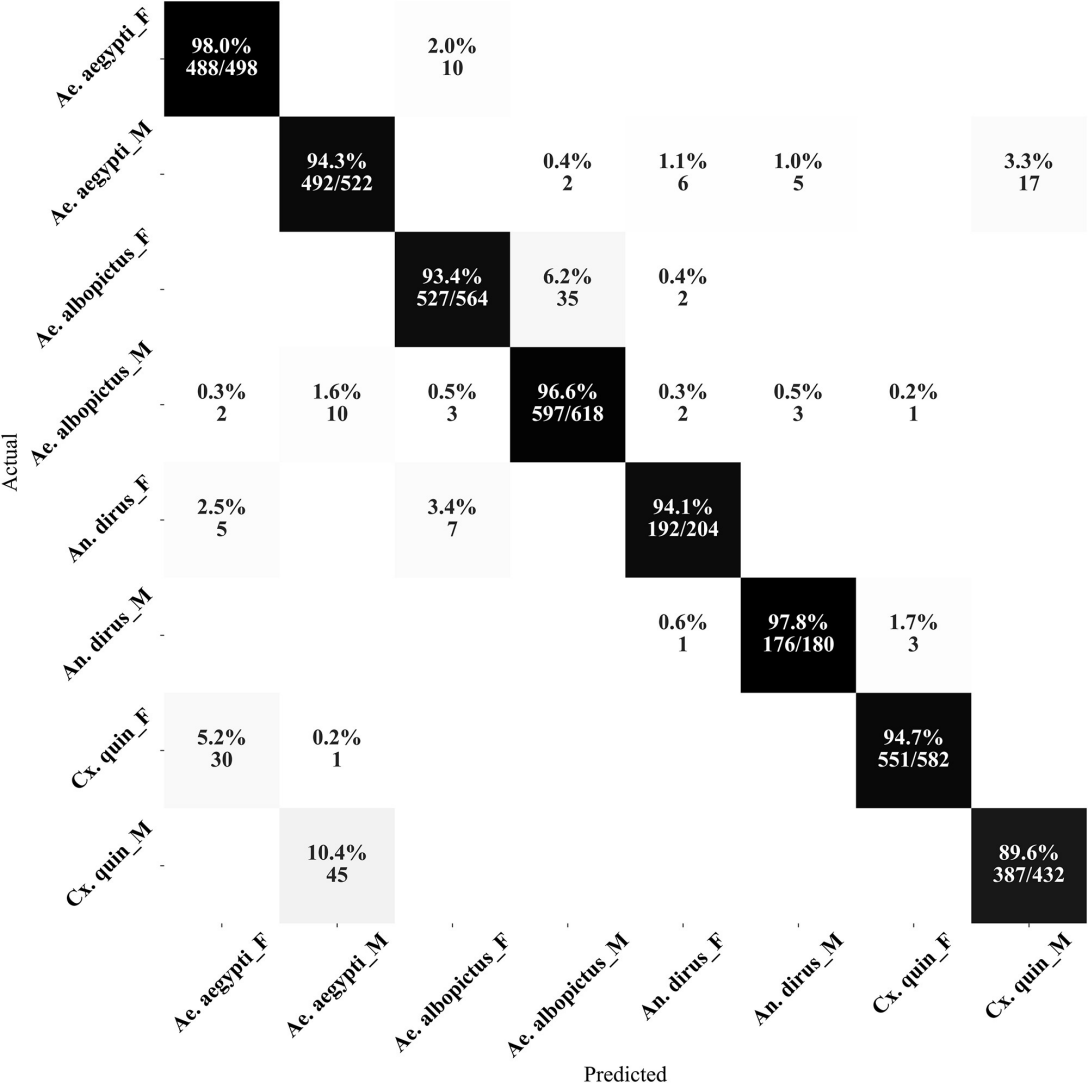
Confusion matrix of our MosquitoSong+ model from one cross-validation fold. Most of the misclassifications were from between *Ae. aegypti* and *Cx. quinquefasciatus* species, which could be due to the overlap between the wingbeat frequency components among these species and sex, with the noise possibly further blurring the distinction. Note: *Cx. quin* refers to *Cx. quinquefasciatus* species.

Upon careful analysis of the confusion matrix of the best method (Fig 3), we observed that most of the misclassifications occurred between *Ae. aegypti* and *Cx. quinquefasciatus* species. This can be attributed to the overlapping wingbeat frequency components between these species and sex [41], with the noise potentially adding further ambiguity to the distinction.

#### Impact of different gain factors

As the results were from the combinations of the test sets from three different gain factors (*G*), we further investigate whether the classification performance is influenced by the volume of the wingbeat sounds. Table 4 shows the ACC, MF1, PR, RE and F1 for each of the gain factors (*G*): 1, 1.5 and 2. Our analysis indicates that the volume of the wingbeat sound in the presence of noise has only a minimal impact on classification performance. The overall ACC/F1 score is 0.911/0.902 when G = 1, 0.922/0.915 when G = 1.5, and 0.922/0.916 when G = 2.0. This observation can be attributed, in part, to our data augmentation techniques, which enhance the robustness to variations in the wingbeat volume.

**Table 4:** Species and sex classification performance of our MosquitoSong+ model when tested with different gain factors (*G*): 1, 1.5 and 2 across overall accuracy (ACC), per-class precision (PR), per-class recall (RE), per-class F1-score (F1), and macro-averaged of these metrics (i.e., the Total column). The Total column represents either the total number of examples or the macro-averaged metrics from all species and sex.

Examples	*Ae. aegypti*		*Ae. albopictus*		*An. dirus*		*Cx. quin*		
	F	M	F	M	F	M	F	M	Total
	1660	1740	1880	2060	680	600	1940	1440	12000
*G* = 1 (ACC = 0.911)									
**PR**	0.920	0.855	0.918	0.953	0.834	0.822	0.974	0.913	0.899
**RE**	0.931	0.854	0.893	0.918	0.906	0.925	0.983	0.869	0.910
**F1**	0.925	0.853	0.904	0.935	0.866	0.869	0.978	0.890	0.902
*G* = 1.5 (ACC = 0.922)									
**PR**	0.908	0.860	0.935	0.968	0.884	0.871	0.980	0.909	0.914
**RE**	0.952	0.869	0.896	0.937	0.897	0.917	0.987	0.886	0.918
**F1**	0.928	0.863	0.915	0.952	0.888	0.891	0.983	0.897	0.915
*G* = 2 (ACC = 0.922)									
**PR**	0.884	0.868	0.936	0.973	0.892	0.893	0.976	0.908	0.916
**RE**	0.958	0.871	0.874	0.945	0.891	0.913	0.989	0.897	0.917
**F1**	0.919	0.868	0.904	0.959	0.890	0.901	0.982	0.902	0.916

*Cx. quin* refers to *Cx. quinquefasciatus* species.

### Experiment 2: Model generalizability—Classification of species across different datasets

#### Experimental setup

To demonstrate the model’s generalizability across a variety of data characteristics, we conducted both training and testing on a combined wingbeat dataset composed of all three datasets: W-INDOOR, W-OUTDOOR, and W-HUMBUG. However, due to incomplete sex annotations in some species within W-HUMBUG, our evaluation and comparison of model performance is limited to mosquito species classification.

Prior to merging these datasets, we independently divided each of them into three distinct subsets: training, validation, and test sets. Subsequently, we combined the training portions from the datasets to train the model. To address imbalances within the training set, we applied the random over-sampling method separately to each class within each dataset before merging them. The noise overlay simulation was only applied to the W-INDOOR and W-HUMBUG datasets, not W-OUTDOOR, as they were not recorded with any background noise. The noise recordings utilized in this experiment were from the N-OUTDOOR and N-HUMBUG datasets.

During the training, we applied our data augmentation techniques to the W-INDOOR and W-HUMBUG datasets. In contrast, the W-OUTDOOR dataset underwent augmentation using only the techniques previously employed in our earlier work [34], which do not involve noise augmentation. The combined validation sets were used in selecting the best performing model during the training phase.

During testing, the noise simulation was only applied to the W-INDOOR and W-HUMBUG datasets. Similar to Experiment 1, we also kept the original wingbeat sounds in the test set of each dataset, such that there were both the wingbeat with and without background noise. It is realistic to assume that there would be periods of wingbeat with and without background noise. The test set from the W-OUTDOOR dataset was directly used for evaluation, as they already contained background noises from the real environment.

It is important to note that during the experiment, there was no overlap in mosquito and environmental noise recordings among the training, validation, and test sets. This was done to ensure that the evaluation of the model reflects a real-world scenario where the model is exposed to wingbeat recordings from different mosquitoes and background noises.

#### Species classification performance

Our model performed well on all datasets with overall ACC and MF1 scores exceeding 0.80 and 0.79, respectively (see Table 5). Specifically, the overall ACC/MF1 score was 0.893/0.873 for W-INDOOR, 0.825/0.807 for W-HUMBUG, and 0.805/0.791 for W-OUTDOOR. Notably, the dataset that exhibited the highest performance was the W-INDOOR, while the lowest performance was observed in the W-OUTDOOR. The species with the highest F1-score consistently was *Cx. quinquefasciatus*, while the species with the lowest score varied across datasets. These variations in performance may be attributed to differences in recording hardware and environmental conditions among the datasets. Such disparities may have led to variations in wingbeat sound patterns, presenting challenges for the model in accurately identifying distinguishing features.

**Table 5:** Species classification performance of MosquitoSong+ evaluated on test sets from W-INDOOR, W-HUMBUG, and W-OUTDOOR datasets in terms of overall accuracy (ACC), macro-averaging F1-score (MF1), and per-class F1-score. For the W-INDOOR and W-HUMBUG datasets, the performance metrics were evaluated on an independent test set with and without noise overlay simulation, each corresponding to the application of the three gain factors (*G*): 1, 1.5, and 2. The W-OUTDOOR dataset was tested directly without the simulation.

	ACC	MF1	Per-class F1-score				
			*Ae. aegypti*	*Ae. albo*	*An. dirus*	*Cx. quin*	Examples
**W-INDOOR** [34]							
*G* = 1	0.883	0.863	0.855	0.908	0.759	0.931	1200
*G* = 1.5	0.896	0.876	0.862	0.927	0.781	0.936	1200
*G* = 2	0.901	0.881	0.872	0.929	0.783	0.94	1200
**Total**	0.893	0.873	0.863	0.921	0.774	0.936	3600
**W-HUMBUG** [35]							
*G* = 1	0.827	0.805	0.846	0.696	0.813	0.865	324
*G* = 1.5	0.821	0.803	0.848	0.727	0.773	0.865	324
*G* = 2	0.827	0.812	0.839	0.75	0.787	0.873	324
**Total**	0.825	0.807	0.844	0.724	0.791	0.868	972
**W-OUTDOOR**							
**Total**	0.805	0.791	0.736	0.675	0.873	0.881	195

*Ae. albo* refers to *Ae. albopictus* species, and *Cx. quin* refers to *Cx. quinquefasciatus* species.

**Fig 4:**
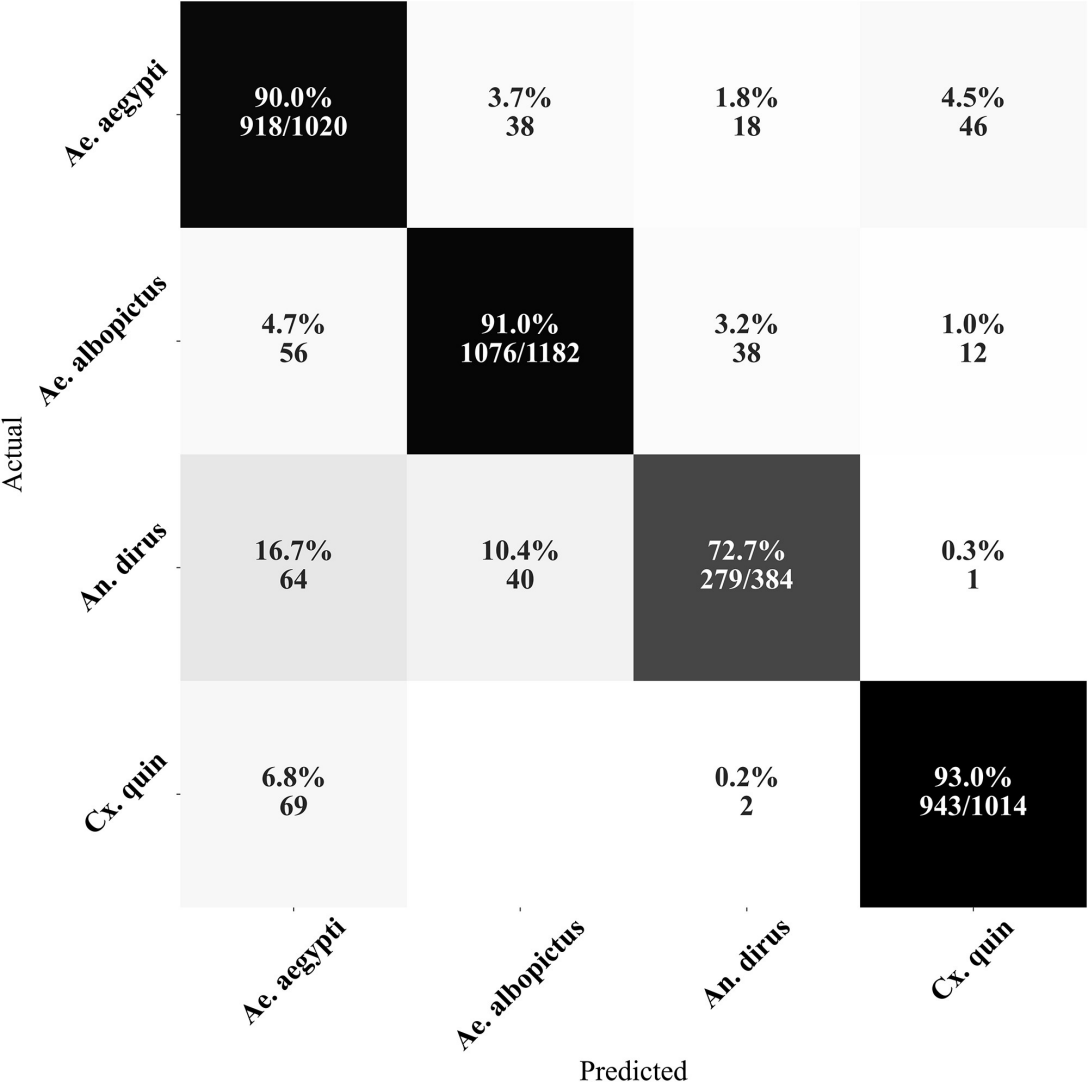
Confusion matrix of the species classification from W-INDOOR. The confusion matrix indicates that the majority of the misclassifications were due to *An. dirus* being misclassified as *Ae. aegypti*. Note: *Cx. quin* refers to *Cx. quinquefasciatus* species.

**Fig 5:**
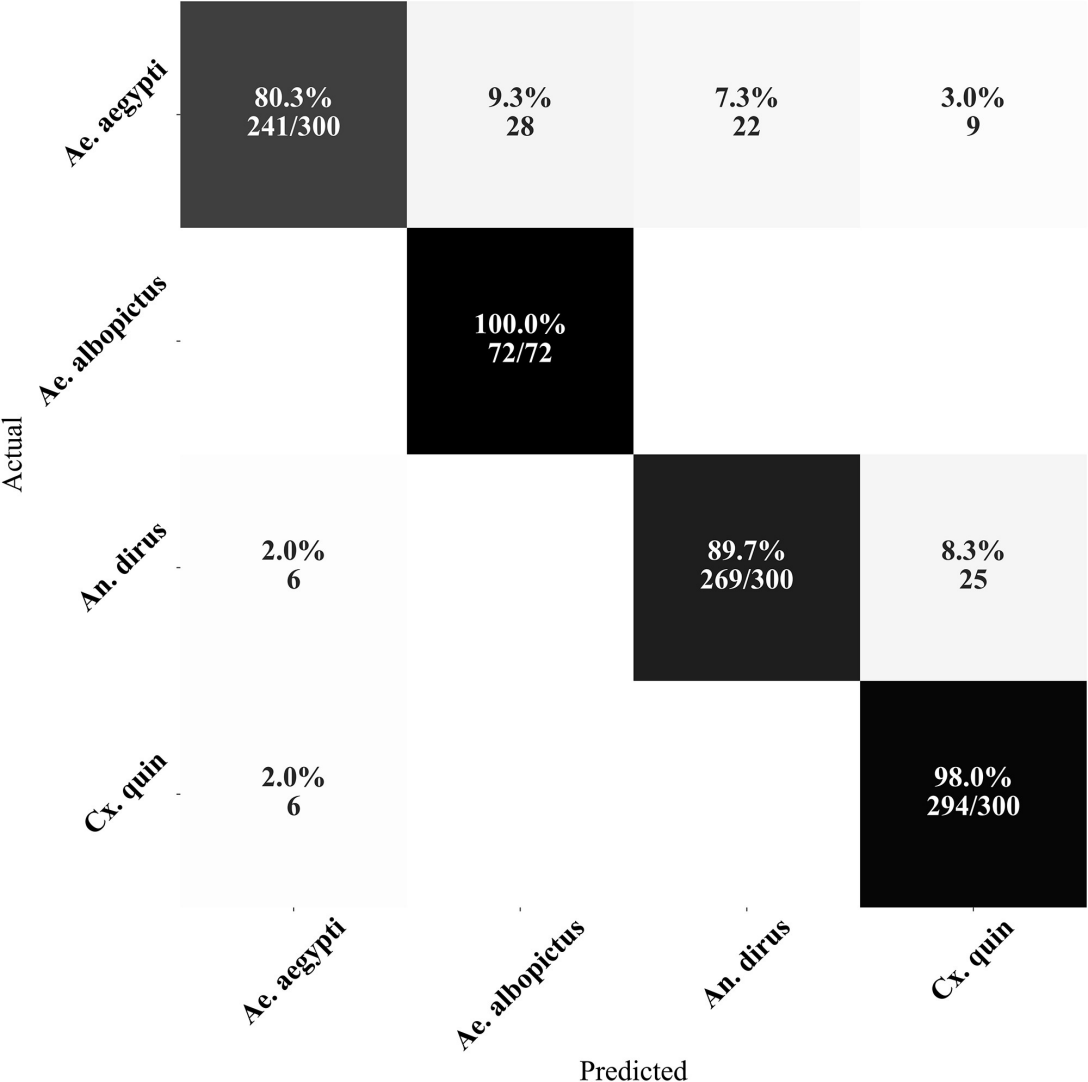
Confusion matrix of the species classification from W-HUMBUG. The confusion matrix indicates that the majority of the misclassifications were due to *An. aegypti* being misclassified as *Ae. albopictus*. Note: *Cx. quin* refers to *Cx. quinquefasciatus* species.

**Fig 6:**
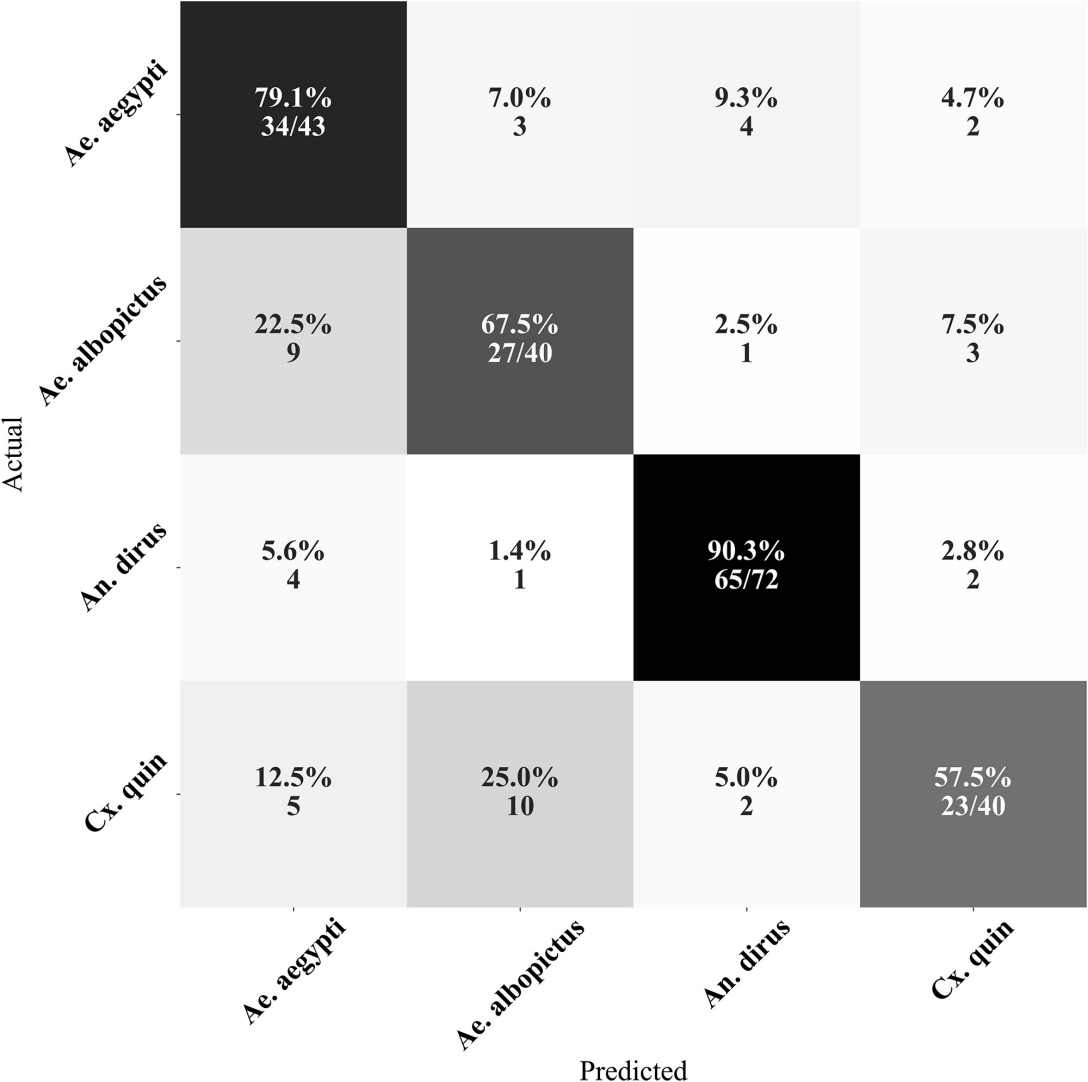
Confusion matrix of the species classification from W-OUTDOOR. The confusion matrix indicates that the majority of the misclassifications were due to *Cx. quin* being misclassified as *Ae. albopictus*. Note: *Cx. quin* refers to *Cx. quinquefasciatus* species.

After analyzing the confusion matrices of each dataset, it was found that the primary reason for misclassification is the confusion between the Aedes genus and other genera. On the W-INDOOR dataset, the misclassification was mostly due to *An. dirus* being misclassified as *Ae. aegypti* (Fig 4). On the W-HUMBUG dataset, the largest misclassification error was due to *Ae. aegypti* being misclassified as *Ae. albopictus* (Fig 5). On the W-OUTDOOR dataset, the main misclassification error was *Cx. quinquefasciatus* being misclassified as *Ae. albopictus* (Fig 6).

Upon analyzing the model performance across all three different *G* values on various datasets, we observed that the amplitude of mosquito sounds relative to background noises had minimal impact on the model’s performance. This finding aligns with the results obtained in Experiment 1, indicating that our data augmentation techniques effectively mitigate variations in wingbeat amplitude within noisy environments.

### Experiment 3: Model performance in practice—Classification of species and sex under simulated vs. real noisy environments

#### Experimental setup

To investigate the MosquitoSong+ model’s ability to determine mosquito species and sex in real noisy environments further, we compared the classification performance between the W-INDOOR and W-OUTDOOR datasets. The experimental setup closely resembled that of Experiment 1. Specifically, the two datasets were combined before being used to evaluate the model using stratified 10-fold cross-validation. There was no overlapping between the training, validation, and test sets in each fold for both wingbeat and environment noises.

For the W-INDOOR dataset, we maintained the original wingbeat sounds in the test set, such that there were both the wingbeat sounds with and without background noise. Three gain factors (G): 1, 1.5, and 2 were applied, resulting in three separate 10-fold cross-validations. However, there was one difference between the simulation of W-INDOOR and Experiment 1. Both the N-OUTDOOR and N-HUMBUG datasets were employed to simulate noisy environments. This is because we would like the classifier to be robust to a wide range of environmental noise. For the W-OUTDOOR dataset, the recordings were used as-is, collected in real noisy environments, and the 10-fold cross-validation was conducted once.

#### Comparison of species and sex classification performance

The results (see Table 6) indicate that the inclusion of N-HUMBUG and W-OUTDOOR datasets contributed to an enhancement in species and sex classification performance, measured in terms of ACC/MF1, on the W-INDOOR dataset compared to Experiment 1 (i.e., 0.933/0.928 vs. 0.918/0.911). This improvement is likely attributed to the model’s exposure to a broader range of wingbeat patterns in the presence of more diverse noises from both simulated and real environments. Similar to the findings in Experiments 1 and 2, the model performance was minimally impacted by the different gain factors, resulting in ACC/MF1 scores of 0.927/0.921, 0.935/0.930, and 0.936/0.933 when G was set to 1, 1.5, and 2, respectively.

**Table 6:** Comparison of Species and sex classification performance of MosquitoSong+ between the simulated (W-INDOOR) and the real (W-OUTDOOR) noisy environments in terms of overall accuracy (ACC), macro-averaging F1-score (MF1), and per-class F1-score. For the W-INDOOR dataset, the performance metrics were evaluated on an independent test set with and without noise overlay simulation, each corresponding to the application of the three gain factors (*G*): 1, 1.5, and 2. The W-OUTDOOR dataset was tested directly without the simulation.

	ACC	MF1	Per-class F1-score								
			*Ae. aegypti*		*Ae. albopictus*		*An. dirus*		*Cx. quin*		Examples
			F	M	F	M	F	M	F	M	
**W-INDOOR** [34]											
*G* = 1	0.927	0.921	0.935	0.877	0.925	0.943	0.901	0.891	0.980	0.919	12000
*G* = 1.5	0.935	0.930	0.938	0.884	0.932	0.948	0.921	0.911	0.986	0.923	12000
*G* = 2	0.936	0.933	0.937	0.887	0.932	0.949	0.927	0.917	0.987	0.927	12000
**Total**	0.933	0.928	0.936	0.883	0.929	0.947	0.916	0.906	0.984	0.923	36000
**W-OUTDOOR**											
**Total**	0.673	0.611	0.641	0.434	0.614	0.324	0.732	0.862	0	0.671	1950

*Cx. quin* refers to *Cx. quinquefasciatus* species.

Conversely, the performance of species and sex classification on the W-OUTDOOR dataset (i.e., ACC/MF1 = 0.673/0.611) did not match the species classification results in Experiment 2 (i.e., ACC/MF1 = 0.805/0.791). Among all the genera, *Anopheles* showed the best F1-score (i.e., F1-score between 0.73 and 0.86) while *Aedes*, especially the male, showed the worst F1-score (i.e., F1-score between 0.324 and 0.614). This decrease in performance may be attributed to three main factors. First, the species and sex classification is a more difficult task compared to the species classification. Second, the distribution of samples across species is imbalanced (see Table 1). Despite employing the data and noise augmentation during model training, the results suggest that the augmented data may not generate adequate variation in wingbeat patterns from a limited number of samples to accurately represent the diverse range of wingbeat patterns encountered in practice. Lastly, there was an imbalance in the number of male and female samples within the same species. For instance, the ratio between the number of epochs of males and females for *Ae. aegypti* and *Ae. albopictus* was approximately equal to or more than 50%. Although the model performed well in species classification for *Cx. quinquefasciatus* in Experiment 2, there was a significant drop in species and sex classification due to the absence of female species in the W-OUTDOOR dataset. These findings suggest that collecting more wingbeat examples from the target environments could substantially improve model performance.

## Discussion

With the aim of achieving a classification model that is robust to a variety of background noise, this study has presented an approach that combines an architecture that improves upon the existing MosquitoSong model, along with data augmentation techniques that incorporate noise, as well as its volume relative to the wingbeat sounds. The results from Experiment 1 show that the MosquitoSong+ model achieves significantly better species and sex classification performance than the previous MosquitoSong model in the presence of noise. After evaluating the performance of the model across different datasets in Experiment 2, we observed that it is capable of classifying species with high accuracy even in noisy environments, whether simulated or real. These environments include various recording hardware and environmental conditions, which demonstrates the model’s versatility. In the final experiment, additional noise was added from both N-OUTDOOR and N-HUMBUG datasets to the simulated noisy environment. Surprisingly, the model performed even better than in Experiment 1. This could be due to the additional variation of wingbeat sounds from the real noisy environment. However, we noticed a performance gap between the simulated and actual noisy environments. Specifically, the model showed suboptimal performance in species and sex classification in real noisy conditions. This is likely due to the limited sample size and imbalanced class distribution within the W-OUTDOOR dataset.

### Data augmentation in mosquito wingbeat classification

Even though data augmentation that introduces variation of the original signals [40] and background noises [24] has already demonstrated its potential in bird species identification in noisy environments, there remains the question of whether such data augmentation is effective in mosquito wingbeat classification. This study is the first to evaluate and demonstrate the effectiveness of data augmentation that introduces the presence of noise and the relative volume of the wingbeat sound in mosquito wingbeat classification based on raw audio signals. Also, as this method trained the model based on the raw audio waveform, another interesting technique that can be incorporated into the model is band-pass filtering [36, 43]. This can be used to filter out frequency components that are, for instance, irrelevant to the mosquito hearing [9] during a pre-processing step before the model training and prediction. The CNN model can then focus more on learning the filters that are useful for distinguishing species and sex, instead of the noise. It can also be used to filter out human voices to address privacy concerns.

### Compared to existing works

In contrast to other work on bioacoustic systems to monitor the general mosquito density in the field by detecting periods of wingbeat sounds in audio streams [19, 35], our study focuses on the classification task (i.e., identifying species and sex). The HumBug Project [19], for instance, has developed a Bayesian CNN model (extended from [21]) to detect mosquito wingbeat sounds based on the Mel-frequency spectrogram representation of the audio recordings. They were able to distinguish mosquito wingbeat sounds from background noise with 89% accuracy in 7.1 hours of field recordings. However, the main difference between their study and ours is that they did not have noise mixed in with the wingbeat sounds; rather, the sounds were played consecutively. A 2D CNN model with a similar architecture was also developed for the species classification task on field recording [18]. They achieved an average classification accuracy of 60% across six species + no mosquito. In comparison, our study achieved an average classification accuracy of 82.5% across four species using the same dataset. To achieve the full functionality that is needed to monitor mosquito vector populations, future work should focus on integrating the best models for detection with those for classification or developing single end-to-end models.

### Impact of wingbeat variations

The species classification performance of W-INDOOR presented in Experiment 2 was not as high as the species and sex classification performance in Experiment 1 and 3, even though it is an easier task. This decline in performance may well be due to the significant variations in wingbeat patterns in W-HUMBUG, which were introduced by discrepancies in recording configurations, hardware, environments, and mosquito origins. Despite the lower species classification performance of the W-INDOOR dataset, our model still maintained an accuracy (ACC) and macro F1 score (MF1) higher than 0.8 across all three wingbeat datasets, demonstrating its generalization capabilities to other datasets.

### Noise cancellation via dual-microphone signal subtraction

We also investigated whether dual-microphone signal subtraction for noise cancellation could reduce the impact of noise on species and sex classification performance. This approach assumes that the source of the desired sound is closer to one microphone than to the other. The envisaged configuration is that one microphone will be located close to the entrance of a mosquito trap, with the second microphone 30 cm away from the trap. In this way the one close to the trap should pick up the wingbeat sound from a flying mosquito much louder than the other microphone. In contrast, the background noise coming from a farther distance will reach both microphones in approximately equal volume.

We compared the model performance using a setup similar to Experiment 1, between noise overlay simulations with and without noise cancellation. We found that noise cancellation had no effect on the performance of the MosquitoSong+ model. The ACC/MF1 were the same with and without noise cancellation: 0.918/0.911 vs. 0.918/0.911. This may be because the data augmentation techniques already provided a high level of robustness to background noise. However, we believe that noise cancellation may help reduce the impact of environmental noises not included in the model training data.

### Limitation

Even though our results are encouraging, the proposed method is still subject to several limitations. First, the datasets used in the study did not control for the temperature, humidity, or age of the mosquitoes. However, research has shown that factors like mosquito age and environmental conditions, including temperature and humidity, have an impact on wingbeat frequencies [19]. Thus, it would be important to conduct further data collection and to add to the model environmental factors like temperature that can be sensed and used as input at inference time. Secondly, our data augmentation techniques rely on the availability of environmental noise datasets, with only two noise datasets utilized in this study. To address this limitation, deploying acoustic sensors in the field to record noises from the target location could facilitate the acquisition of additional noise data, which can be done at the beginning of the surveillance process. Subsequently, the model can be calibrated with noises specific to the target location. Third, while our data and noise augmentation techniques helped introduce variation of the mosquito wingbeat patterns with various background noises, they may not fully account for the actual variation in wingbeat patterns observed in practice. As the amount mosquito recordings from real noisy environments from different species and sex is limited, further studies are necessary to validate the model performance when more wingbeat examples become available.

In the future, we plan to evaluate the MosquitoSong+ model in a low-cost IoT device to evaluate its effectiveness in the real field environment. This includes the investigation of how to incorporate the proposed model into a pipeline for mosquito detection and classification from wingbeat sounds [41], how to calibrate or fine-tune the model after the initial data collection of the environmental noises at a target location, and how to fuse the features from acoustic and optical sensors for the classification. We also plan to study how to combine the mosquito counts from the IoT devices with the density maps of the potential vector breeding sites from geotagged images [44] to improve the estimation of vector populations. We aim to incorporate such estimates into predictive models to improve their accuracy, since current models typically rely on proxies to estimate mosquito vector populations [45].

## Conclusion

We presented the *MosquitoSong+* model to identify mosquito species and sex from wingbeat sounds in the presence of environmental noise. Our experimental results, which included noise overlay simulations, indicate that the new 1D-CNN architecture, along with two data augmentation techniques, significantly improved the model’s performance in species and sex classification. Furthermore, our results demonstrate the model’s generalizability, achieving accuracy above 80% in species classification across various datasets, including indoor wingbeats with noise overlay, HumBugDB wingbeats with noise overlay, and outdoor wingbeat recordings. Even though the initial results of the species and sex classification on the outdoor recordings were less accurate than the noise overlay simulation, this is likely due to the limited and imbalanced number of examples used. Therefore, further study is required to validate the model performance on a larger number of outdoor recordings. Future work should focus on integrating the detection and classification models to create a comprehensive mosquito monitoring system. Additionally, field evaluations are crucial to validate the effectiveness of MosquitoSong+ and to identify practical challenges that may arise in deploying the approach in real-world settings outside of controlled laboratory environments.
